# Perioperative Anaphylactic Risk Score For Risk-Oriented Premedication

**Published:** 2013-09-02

**Authors:** Giacomo Manfredi, F. Pezzuto, A. Balestrini, M. Lo Schiavo, M.C. Montera, A. Pio, M. Iannelli, D. Gargano, M.J. Bianchi, G. Casale, M. Galimberti, M. Triggiani, O. Piazza

**Affiliations:** 1 Religious General Hospital “F. Miulli”, Acquaviva (BA), Italy; 2 Casa di Cura Tortorella S.p.a. Salerno, Italy; 3 Azienda Ospedaliera Universitaria Ruggi d’Aragona, Università di Salerno, Salerno, Italy; 4 Azienda Ospedaliera G. Moscati di Avellino, Italy; 5 Fleming Research SRL, Novara, Italy

**Keywords:** anaphylactic risk, perioperative, anaesthesia

## Abstract

Basing on the current knowledge, this paper is aimed to review the core characteristics of the most relevant therapeutic agents (steroids and antihistamines), administered to prevent perioperative anaphylaxis. Moreover, the Authors propose the validation of a Global Anaphylactic Risk Score, built up by recording the individual scores related to the most relevant anaphylaxis parameters (i.e. medical history, symptoms and medication for asthma, rhinitis and urticaria etc) and by adding them on all together; the score could be used in the preoperative phase to evaluate the global anaphylactic risk and to prescribe risk-oriented premedication protocols.

## I. INTRODUCTION

More and more, the allergologists have been involved, together with the anaesthesiologists, in the perioperative anaphylactic risk evaluation and decision making about which premedication drugs should be prescribed. These decisions are difficult because of the intrinsic complexity of diagnostic procedures and the frequent comorbidities (i.e. diabetes, arterial and/or ocular hypertension, cerebral angiomas, immune deficiencies, etc) and the poor diffusion of guidelines. Consequently we propose a tool to evaluate Global Anaphylactic Risk Score that is aimed to: 1) to ensure a correct diagnostic and patient-oriented therapeutic procedure; 2) to attain an elevate level of procedures standardization, in order to avoid personalized therapeutic drifts; 3) to optimize recovery times in the case of adverse reactions; 4) to reduce legal problems related to incorrect therapeutic procedures for the patient’s recovery. Anaphylaxis is the heaviest generalized immediate hypersensitivity reaction, potentially life-threatening, marked by the release of multiple chemical mediators and cytokines, having multiple effects on metabolism, cardiovascular and respiratory systems and virtually all organs. Although it was firstly described one hundred years ago, until now the agreement about its definition and diagnostic criteria has not been reached. Nowadays, the term of “anaphylactoid reaction” has been abandoned and all the generalized reactions are classified as anaphylaxis; the latest document of World Allergy Organization [ 1 ] distinguishes the following pathogenic mechanisms of anaphylaxis:

**Immunologic**

1. IgE mediated (Type-I of Gell and Coombs classification) i.e. foods, drugs, hymenoptera venom, etc.
2. Not IgE-mediated (involvement of IgG and/or complement) (Type II, mediated by antibodies and/or complement, and III, by immune complexes); i.e. radio contrast agents, biological agents, blood products, drugs.

**Not Immunologic:** due to direct mast cells activation (because of physical causes: exercise, cold temperature, or ethyl alcohol and others drugs)

**Idiopathic:** mast cells clonal disorders, unknown causes. The prevalence of perioperative anaphylactic reactions, IgE or not-IgE mediated, has increased in the last decades because of the wide diffusion of surgical procedures; these reactions can be due to Anaesthetics, Muscle Relaxant, Hypnotics or Latex. Notwithstanding the large differences in epidemiologic studies, it can be assumed that the prevalence of perioperative anaphylactic reactions is about 1/13000 anaesthesia procedures globally considered (local, regional, general anaesthesia). The mortality attributed to perioperative anaphylaxis ranges from 3,5 to 9 % [ 2 ] . As muscle relaxants are concerned, succinilcolyne, rocuronium, atracurium, vecuronium, pancuronium, mivacurium and cisatracurium are the main responsible agents for anaphylactic reactions. Curarics [ 3 ] have the highest anaphylactic reactions rate (50–75% out of all reactions); this is caused by the high content in quaternarium ammonium ions, acting as antigenic determinants able to bind to specific IgE. As to hypnotics, both barbiturics (thiopentale) and non barbiturics (propofol) may induce hypersensitivity reactions with IgE as well as non IgE-mediated mechanism. Anaphylactic reactions during general anaesthesia may supervene because of IgE-mediated sensitization to ethylene oxyde or to plasma expanders (polygelines). Latex might be responsible for more than 10% of intraoperative anaphylactic reactions because of contact with gloves, catheters and endotracheal tubes.

### PREMEDICATION

The drugs able to antagonize histamine effects (antiH1, antiH2) and those modulating immune system activation (steroids) are the most used to prevent the anaphylactic reactions induced by anaesthetics. In the latest years many different premedication protocols for patients at risk for anaphylaxis have been proposed; so far, these protocols [ 4 ] induce some perplexities because of the side effects of high dosage steroids, their lymphocytolytic and immunosuppressive effect, the frequent presence of comorbidities, and the fact that the same fixed dosage is prescribed for all the patients. Various combination of anti-H1, anti H2 and glucocorticoids are used with different time schedule and way of administration ( *per os* or i.m.).

### CORTICOSTEROIDS

When an antinflammatory or immunosuppressive effect is desirable, steroids having low mineralcorticoid and high glucocorticoid action, as prednisone, prednisolone, methylprednisone, desametasone, betametasone are the most used.

Glucocorticoids (GCs) are potent anti-inflammatory and immunosuppressive agents, which exert multiple effects on immune cell functions. GCs act through different genomic and non-genomic mechanism, mediated by their binding to cytosolic glucocorticoid receptor or by interacting directly with enzymes and other cell proteins. GCs actions can be early or late: their early effect is described as “non genomic” and it’s limited to very high doses administration; the late effects is described as “genomic”, it takes more time (it requires longer than 24–48 hours) in order to realize the link between the drug and the cytoplasmic receptor (GREs) and the subsequent translocation of the complex steroid-receptor to the nucleus; this complex then reacts with specific genomic sequences influencing the mRNA transcription encoding enzymes inhibiting the synthesis of inflammation mediators.

The latter mechanism requires several hours and then cannot be invoked to justify the clinical use of GCs for the immediate prophylaxis and treatment of anaphylaxis while the non-genomic actions justify the rapidity action of GCs administrated in high doses ( tab 2 ). GCs are able to interact directly with the cell membrane entering the phospholipidic double-layer, altering its properties. This effect can also occur in the mitocondria, inducing a proton conductance abnormality, which causes a lower production of ATP. Another non-genomic mechanism consists in the release of heat shock protein (HSP) and chaperonins by the GC-GCR complex, leading to release of arachidonic acid from membrane phospholipids. GCs can bind to another type of receptor for GC expressed on the cell membrane (GCm). In recent years, the link between GCs and post-transcriptional mechanisms has been remarked in many studies. The control of posttranscriptional pathways could exert rapid anti-inflammatory actions since post-transcriptional effects take few minutes to become evident, as shown on human epithelial airway cells [ 5 ] .

The link between GCs and post-transcriptional effects can be so schematized:

1. GCs accelerate the mRNA decay of cytokines (IL-6, IL-1α, etc.) chemokines and pro-inflammatory molecules.
2. GCRs act as RNA-binding protein.

For these reasons and by these pathways, GCs can turnoff the basal activity of some immune system cells before genomic effects.

The genomic action is attained also with lower doses of the drug; so far it hasn’t been yet established a precise dose-response relationship between genomic and nongenomic action. Anyway it has been pointed out that the needed dose to attain a sharp non-genomic effect is higher than 100 mg of prednisone or another equivalent glucocorticoid. The steroids gained an undoubtedly preeminent place in enhancing the bronchial beta2 receptors sensitivity to beta-agonists, so that they are unchangeable drugs to attain asthma control; these effects are linked to genomic mechanisms that need the right time to switch on. On the other hand, also the inhibition of glucocorticoids on a wide range of T and weakly B lymphocyte immune responses, as well as the powerful suppressive effect on phagocyte functions, require a variable latent period related to the intrinsic action mechanism. On the contrary, the efficacy of steroids in inhibiting the mast cells chemical mediators release is just partial; many authors, working on isolated pulmonary, intestinal and cutaneous mast cells [ 6 ] , showed that desametasone is not able to inhibit preformed mediators release; that’s the reason why the steroid efficacy in the anaphylaxis treatment is really limited to the late phase and doesn’t work in the early phase. Moreover, there are other in vivo experiences confirming this point of view: Naitoh et Al reported that high dose steroid therapy was not able to inhibit systemic anaphylaxis after cotrimoxazole in a patient suffering from Lupus Erythematosus Systemicus [ 7 ] . A recently published Cochrane systematic review [ 8 ] about the use of glucocorticoids in the anaphylaxis treatment showed that there aren’t highly quoted published papers from 1945 till today in favour of the glucocorticoids efficacy in the emergency treatment of anaphylaxis.

### ANTIHISTAMINES

H-1 antihistamines act as histamine receptor reversible antagonists; they are generally well absorbed by gastroenteric tract and after oral taking they reach the plasmatic concentration peak and start their action in average 45–120 min; the pharmacologic effects last from 4–12 to 24 hours and in the case of particular molecules, a few days ( tab 1 ).

Because of these peculiar characteristics, H1 antihistamines, alone or in combination with antiH2, have been used in many premedication protocols in patients at risk of adverse reaction to general anaesthetics. H1 antihistamine can prevent the oedema due to histamine-induced capillary hyperpermeability and inhibit hyperemia and itch. These drugs inhibit both histamine-mediated vasoconstriction and partially its early vasodilatory effects, mediated by H1 receptors placed on endotelial cells. Residual vasodilation is imputable to vessel musculature H2 receptors involvement and can be inhibited by co-administration of H2 antihistamines. The premedication with H1 antihistaminees isn’t sufficient to prevent an adverse reaction [ 9 ] ; this unmet need has been observed both in latex and drugs allergies [ 10 ] . The inefficacy in preventing not specific histamine release induced bronchospasm and hemodynamic alterations [ 11 ] , together with considerations regarding drugs pharmacokinetics, limit the utility of the administration of anti H1 before general anaesthesia in patients at risk, when this is aimed to reduce or impair possible adverse reaction.

The doubts and uncertainties regarding the efficacy of premedication with antihistamines are linked to the consideration that there is no certainty that the full receptors’ saturation can be achieved, particularly at the respiratory level, following the doses, ways and drugs formulations used; consequently the antihistamine premedication should be started at least three days before the event, doubling the daily dose and using the last generation drugs securing higher action speed and reducing the main side effects as sedation. Moreover, we can’t forget pharmacokinetic and pharmacodynamic considerations imposing parenteral administration when an immediate effect is desirable.

## II. PREOPERATIVE ANAPHYLACTIC RISK EVALUATION

**Fig. 1:**
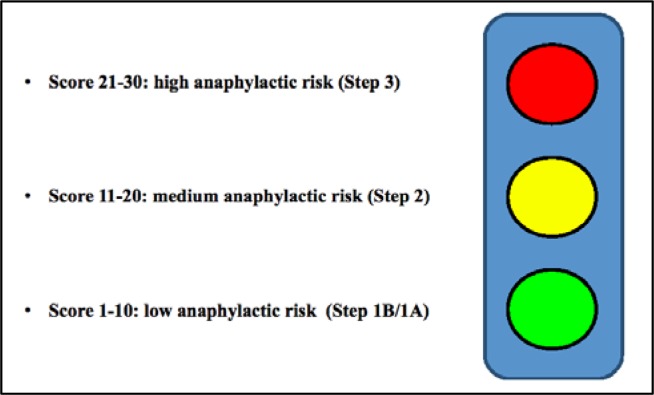
Traffic light (color code) exemplification of GARS

**Fig. 2:**
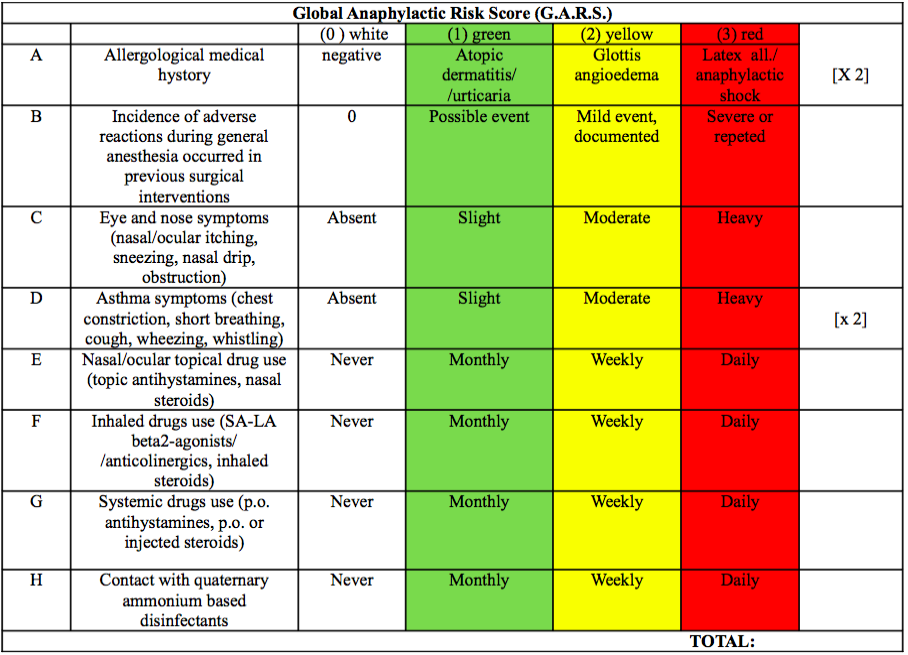
This figure illustrates an attempt to calculate GARS. Risk parameters may be implemented and accuracy of correction factor needs to be tested. Green is equal to 1 point, red sums up 3 points, white is no significant risk.

We propose a tool to mark some critical parameters regarding the preoperative anaphylactic risk. The Global Anaphylactic Risk Score (G.A.R.S.) can be represented as traffic-light symbol on the basis of colour (the risk can be described as white, green, yellow, red, following the colour code used in the ER triage, fig.1 .) The score sums up 8 parameters, obtained from scientific literature and openly discussed among the Authors. Other parameters may be relevant but a larger study is needed to retrieve them properly. In our preliminary example, the parameters are multiplied by an arbitrary (that means decided by the Authors, basing on their experience and needing scientific validation through a focused clinical study) correction factor (as shown in the last column, figure 2 ), to attain a global score expressing the anaphylactic risk.

Cardiac ischemia and arrhythmias, i.e. are considered severe risk factors because the subsequent hemodynamic alterations are able to worsen the clinical outcome of a possible anaphylaxis. Moreover the rich mast cells representation at cardiac level and arrythmogenic effects of the histamine released during allergic reaction justify the risk increase in these clinical situations. When drugs and/or latex allergy or a documented previous adverse reaction to general anaesthetics are reported, an allergologist evaluation is strongly advised.

The systemic mastocytosis requires a separate mention; as well known, this diagnosis is based on clinical (peculiar skin alterations), hystologic (parenchymal mast cells infiltration) and laboratory (blood tryptase increase) criteria; mastocytosis affects the anaphylactic risk and its therapy must be decided in agreement with oncohematologists.

## III. RISK-ORIENTED PREMEDICATION PROTOCOLS FOR SCHEDULED SURGICAL PROCEDURES

### LOW ANAPHYLACTIC RISK PATIENT: supraglottic involvement only (G.A.R.S.<5): STEP 1A

If the score is equal to or lower than 5, with negative medical history (A=0, B=0) and if there are neither any uncontrolled asthma’s signs (D=0, F=0, G=0), nor frequent use of quaternary ammonium containing disinfectants (H=0-1), but oculo-rhinitis (C=1–2) and occasional assumption of topical drugs, then it could be possible, in our projection which needs confirmation from focused clinical trial, to contain the premedication to NASAL TOPICAL STEROIDS, *per os* H1-ANTIHISTAMINES STARTING 3 DAYS BEFORE THE PROCEDURE AND PROMETAZINE OR CHLORPHENAMINE MALEATE i.m.1 H. BEFORE THE PROCEDURE.

### SKIN INVOLVEMENT (G.A.R.S.<10): STEP 1B

If the score is ranging from 5 to 10, if the patient’s history is positive for skin involvement and/or occasional adverse reactions during previous surgical procedures (A=1×2=2, B=1), then it’s suitable to add H1 ANTIHISTAMINES TO STEP 1 PREMEDICATION, STARTING 1 WEEK BEFORE AND PREDNISONE *per os* 0.5 mg/kg 12 hrs AND 2 hrs BEFORE THE PROCEDURE.

### MEDIUM ANAPHYLACTIC RISK PATIENT WITH MILD AIRWAYS INVOLVEMENT (G.A.R.S.<20): STEP 2

If the attained score is ranging from 10 to 20, i.e. if there is positive anamnesis for glottis angioedema (A=2×2=4), slight/intermittent uncontrolled asthma’s signs, taking into account both symptoms and medication score (D=1–2×2=2–4, F>0, G=1–2), then it could be suitable to add S-LABA PLUS INHALED STEROIDS, H2-ANTIHISTAMINES AND ANTILEUKOTRIENES to step 1B premedication, 2 WEEKS BEFORE THE PROCEDURE.

### HIGH ANAPHYLACTIC RISK PATIENT WITH SEVERE AIRWAYS INVOLVEMENT (G.A.R.S. >20): STEP 3

If the patient has positive anamnesis for latex allergy and/or adverse reactions in previous surgical procedures with high incidence (more than one adverse event out of three anaesthesia procedures), and there are severe uncontrolled asthma’s symptoms (D=3×2=6) and systemic anti-allergic drugs frequent use (G=3), it is necessary to administrate systemic steroid drugs before the procedure to restore sensitivity of bronchial beta receptors to beta-agonists: add prednisone 0.5 mg/kg *per os* daily to step 2 premedication for 2 weeks before the procedure, call the allergologist and activate the latex-free procedure.

## IV. DISCUSSION

In case of emergency-urgency the proposed premedication following the GARS score is not applicable.

The most used premedication in allergic patients before emergency surgery is: 1 mg/kg prednisone i.v., or its equivalent, prometazine i.m. and ranitidine 100 mg i.v. (diluted in saline 100 ml) plus s-aba+inhaled steroids before the surgical procedure. Nevertheless, the efficacy of this premedication protocol is debatable.

Moreover, the asthmatic patient deserves a close attention because she/he’s in all probability prone to peri-operative broncho-constriction’s crises. In case of scheduled surgical procedures, asthmatic patients should be admitted to previous specialist evaluation [ 12 ] at least 1 month before the operation, in order to have a congruous time frame to reach the best possible clinical asthma control [ 13 ] . We recommend performing a careful preoperative research of signs indicating uncontrolled or badly controlled asthma.

At the same time, we think useful to evaluate pulmonary function by O _2_ saturation (SpO _2_ ) and/or arterial blood gas analysis, spirometry with flow-volume curve in asthma patients.

Abnormal spirometric data, evaluated out of patient clinical context, can’t be assumed as absolute contraindication to a non-thoracic surgical procedure; anyway, as precautionary measure, the minimally invasive surgical procedure is preferable when risk is very high.

The preoperative anaphylactic risk’s evaluation is a complex subject always needing the allergologist intervention. This preoperative anaphylactic risk score and the related therapeutic interventions step by step, represent an orientating tool that can’t and must not substitute the clinical judgment of the specialist involved in the clinical reality with all its unrepeatable peculiarity. Moreover, we don’t surely expect to include all possible clinical contexts, especially before a dedicated trial is developed.

## Figures and Tables

**Tab 1:** antihistamines latency and length of action (empty cell are in case of discordant data)

	

	** Latency (hours) **	** length of action (hours) **	** tachiphylaxis **
Desloratadine	<1	24	no
Levocetirizine	<1	24	no
Terfenadine	2	>12	no
Chlorfeniramine	<1	<12	likely
Hydroxyzine		12	no
Fexofenadine	1	12	

**Tab 2:** corticosteroids action in rat models

** drug **	Nakamura (14)	Oliveira (15)
** dose **	Methylprednisolone	Prednisolone
** model **	10 mg/Kg (intra-peritoneal)	0,8–2,4–8 μmol/kg
** time **	Rat; surgical stress mode 1 h before surgical stress	Rat; antigen challenge 1 h before antigen challenge
** effects **	Downregulation at 3 hrs	Reduced cells and inflammation mediators for 24 hrs
